# Thermostable Bacterial Bioflocculant Produced by *Cobetia* Spp. Isolated from Algoa Bay (South Africa)

**DOI:** 10.3390/ijerph9062108

**Published:** 2012-06-04

**Authors:** Anthony Ugbenyen, Sekelwa Cosa, Leonard Mabinya, Olubukola O. Babalola, Farhad Aghdasi, Anthony Okoh

**Affiliations:** 1 Applied and Environmental Microbiology Research Group (AEMREG), Department of Biochemistry and Microbiology, University of Fort Hare, Private Bag X1314, Alice 5700, South Africa; Email: ugbenyenanthony@gmail.com (A.U.); sekco@webmail.co.za (S.C.); lmabinya@ufh.ac.za (L.M.); 2 Department of Biological Sciences, Faculty of Agriculture, Science and Technology, North-West University, Mafikeng Campus, Private Bag X2046, Mmabatho 2735, South Africa; Email: Olubukola.Babalola@nwu.ac.za; 3 Risk and Vulnerability Assessment Centre, University of Fort Hare, Private Bag X1314, Alice 5700, South Africa; Email: FAghdasi@ufh.ac.za

**Keywords:** *Cobetia* sp., OAUIFE, bioflocculant, acidic polysaccharide, flocculating activity, thermostable

## Abstract

A novel bioflocculant-producing bacteria was isolated from sediment samples of Algoa Bay in the Eastern Cape Province of South Africa and the effect of culture conditions on the bioflocculant production was investigated. Analysis of the partial nucleotide sequence of the 16S rDNA of the bacteria revealed 99% similarity to *Cobetia* sp. L222 and the sequence was deposited in GenBank as *Cobetia* sp. OAUIFE (accession number JF799092). Cultivation condition studies revealed that bioflocculant production was optimal with an inoculum size of 2% (v/v), initial pH of 6.0, Mn^2+^ as the metal ion, and glucose as the carbon source. Metal ions, including Na^+^, K^+^, Li^+^, Ca^2+^and Mg^2+^ stimulated bioflocculant production, resulting in flocculating activity of above 90%. This crude bioflocculant is thermally stable, with about 78% of its flocculating activity remaining after heating at 100 °C for 25 min. Analysis of the purified bioflocculant revealed it to be an acidic extracellular polysaccharide.

## 1. Introduction

Flocculants or flocculating agents are chemicals that promote flocculation by aggregation of colloids and other suspended particles, forming a floc [1]. Flocculants are widely applied in various industrial processes, including wastewater treatment, downstream processing, food and fermentation processes [2,3,4]. Although chemical flocculants have been widely used due to their effective flocculating activity and low cost, some synthetic flocculants are known to be hazardous to the environment [5,6,7]. In recent years, utilization of microbial flocculants has been promoted due to their biodegradability and their environmentally inert nature [8,9]. Screening new microorganisms for bioflocculant activity, therefore, has become a subject of intensive investigations globally. 

Marine bacteria are among the most economically and biotechnologically valuable prokaryotes, responsible for the production of about 50% of all discovered bioactive secondary metabolites [10]. The rate of discovery of new compounds from terrestrial bacteria has decreased, whereas the rate of re-isolation of known compounds has increased [11], suggesting the need for exploring underexploited habitats such as the marine environment as sources of novel bioactive compounds including bioflocculants. In this paper, we report a novel bioflocculant produced by a marine bacterium belonging to the genus *Cobetia*, isolated from sediment samples of Algoa Bay in the Eastern Cape Province of South Africa. To the best of our knowledge, this is the first time that the genus has been implicated in bioflocculant production.

## 2. Experimental Section

### 2.1. The Test Bacteria

The test bacteria was isolated from sedimentary samples of Algoa Bay in the Eastern Cape Province of South Africa as part of the culture collections of the Applied and Environmental Microbiology Research Group (AEMREG), University of Fort Hare, Alice, South Africa. The bacterium was maintained in 20% glycerol at −80 °C.

### 2.2. Identification of the Bioflocculant-Producing Bacteria

#### 2.2.1. DNA Extraction

DNA extraction was conducted using the boiling method [12], whereby 2–3 colonies of the test bacteria were suspended in 70 µL of sterile double distilled water. The samples were heated in a water bath at 100 °C for 10 min, allowed to cool for 5 min and thereafter centrifuged at 3,000 rpm for 5 min. The supernatant was transferred to a clean tube and used as template DNA for 16S rRNA gene amplification. 

#### 2.2.2. Polymerase Chain Reaction (PCR) Amplification of 16S rRNA Gene

PCR amplification of the 16S rRNA gene of the test bacteria was carried out in 50 µL reaction volume containing 2 mM MgCl_2_, 2 U Supertherm Taq polymerase, 150 mM of each dNTP, 0.5 mM of each primer (F1: 5'-AGAGTTTGATCITGGCTCAG-3'; I = inosine and primer R5: 5'-ACGGITACCTTGTTACGACTT-3') and 2 mL template DNA [12]. The PCR condition include an initial denaturation (96 °C for 2 min), 30 cycles of denaturation (96 °C for 45 s), annealing (56 °C for 30 s) and extension (72 °C for 2 min), and a final extension (72 °C for 5 min). Gel electrophoresis of PCR products were conducted on 1 % agarose gels to confirm that a fragment of the correct size had been amplified.

### 2.3. Media and Cultivation Conditions

The pre-culture medium consisted of 20 g glucose, 0.5 g urea, 0.5 g yeast extract, 0.2 g (NH_4_)_2_SO_4_, 2 g KH_2_PO_4_, 5 g K_2_HPO_4_, 0.1 g NaCl and 0.2 g MgSO_4_·7H_2_O in 1 L of filtered natural sea water using Whatman filter paper. The initial pH of the medium was 6.3 [13]. Two loopfuls of bacterial colonies were inoculated into 50 mL of the medium and incubated with shaking at 160 rpm for 72 h at 28 °C. Following incubation, 2 mL of the fermentation broth was centrifuged (8,000 g, 30 min) to separate bacterial cells and the cell free culture supernatant was analysed for flocculating activity. The pre-culture was stored at 4 °C and used for subsequent inoculations. Production medium contained the same components as the pre-culture medium [13].

### 2.4. Measurement of Flocculating Activity

Flocculating activity was measured as described elsewhere [2,13,14,15,16], with modifications. Briefly, 3 mL of 1% CaCl_2_ and 2 mL of cell free supernatant were added to 100 mL kaolin suspended solution (4 g/L) in 250 mL flask. The mixture was vigorously stirred and poured into a 100 mL measuring cylinder and allowed to stand for 5 min. The optical density (OD) of the clarifying solution was measured with a spectrophotometer at 550 nm. A control experiment was prepared using the same method, but using fresh culture medium (B). The flocculating activity was calculated according to the equation:

Flocculating activity (%) = [(B − A)/A] × 100

where A is the optical density of the sample at 550 nm; B is the optical density of control experiment at 550 nm. 

### 2.5. Effects of Inoculum Size

Inoculum size is an important parameter in the production of bioflocculant [13,17]. Hence, we assessed the effect of different inoculum sizes on bioflocculant production by the test bacteria. Flasks (150 mL size) containing 50 mL production medium were separately inoculated with 0.5 mL, 1.0 mL, 1.5 mL and 2.0 mL pre-culture of the test bacteria cultivated at 28 °C with agitation at 160 rpm for 72 h. At the end of the incubation period, the fermentation broths were centrifuged (8,000 g, 30 min) to separate the cells and the cell free supernatants were analysed for flocculating activity.

### 2.6. Effects of Carbon and Nitrogen Sources

The effects of organic and inorganic carbon sources on bioflocculant production were assessed. The organic carbon source candidates include glucose, sucrose, fructose, maltose, galactose and xylose; while the inorganic carbon sources include phthalate, sodium acetate and sodium carbonate. Organic nitrogen sources (*viz.*, peptone, tryptone, urea, yeast extract and casein), as well as inorganic nitrogen sources (*viz.*, ammonium chloride, ammonium sulphate and sodium nitrate) were assessed for their effect on bioflocculant production.

### 2.7. Effects of Initial pH and Cations

The effects of initial pH and cations on bioflocculant production were assessed in accordance with the description of Liu *et al.* [9]. The initial pH of the production medium varied in the range of 3–12 using 0.1 M HCl and NaOH, while the cation candidates included Na^+^, K^+^, Li^+^, Mg^2+^, Mn^2+^, Al^3+^ and Fe^3+^ as their chloride salts. With regards to the effects of cation assays, flocculant test were conducted as described above, but CaCl_2_ solution was replaced by a solution of the above cation candidates, and the flocculating activity was measured.

### 2.8. Thermal Stability of the Crude Bioflocculant

The effect of heat on the bioflocculant produced by the test bacteria was assessed according to the method of Gong *et al*. [18], using three different temperature regimes (50 °C, 80 °C and 100 °C). The cell-free culture supernatant (crude bioflocculant) was placed in a waterbath and heated over a period of 25 min. At the end of the 25 min incubation period 2 mL of the bioflocculant was withdrawn and assessed for residual flocculating activity.

### 2.9. Time Course Assay

The medium for the time course assay was composed based on the previously determined optimal growth conditions. Samples (2 mL) were drawn every 12 h over 5 days and centrifuged at (8,000 g, 30 min). The cell-free supernatant was then used to determine the flocculating activity. The optical density (OD) of the broth was also recorded at a wavelength of 660 nm.

### 2.10. Extraction and Purification of Bioflocculant

Extraction and purification was done according to the method described by previous reports [14,15,19,20,21]. After 72 h of fermentation, the culture broth was centrifuged (8,000 g, 30 min) to remove bacterial cells. One volume of distilled water was added to the supernatant phase and centrifuged (8,000 g, 15 min) to remove insoluble substances. To the supernatant, two volumes of ethanol was added, stirred and left to stand for 12 h at 4 °C. The precipitate was vacuum dried to obtain crude bioflocculant. The crude product was dissolved in distilled water to yield a solution, to which one volume of a mixed solution of chloroform and n-butylalcohol (5:2, V/V) was added, stirred and allowed to stand for 12 h at room temperature. Two volumes of ethanol were again added to recover the precipitate, which was then lyophilized.

### 2.11. Analysis of Purified Bioflocculant

Total protein content of purified bioflocculant was determined by Lowry’s method using Bovine Serum Albumin (BSA) as a standard [22]. The total sugar content of bioflocculant was determined by a phenol-sulphuric acid method using glucose as a standard solution [23], and uronic acid was quantified by the carbazole method [24].

### 2.12. Statistical Analysis

Data were analysed by one-way analysis of variance (ANOVA) using MINITAB Student Release 12 statistical package. A significance level of *p* < 0.05 was used. The mean values are of three replication.

## 3. Results and Discussion

Amplification of the 16S rRNA gene of the bacteria resulted in an amplicon of expected size (approximately 1.5 kb). Basic Local Alignment Search Tool (BLAST) analysis of the nucleotide sequence of the 16S rDNA revealed 99% similarity to that of *Cobetia* sp. L222 and the nucleotide sequence was deposited in GenBank as *Cobetia* sp. OAUIFE with accession number JF799092.

**Figure 1:**
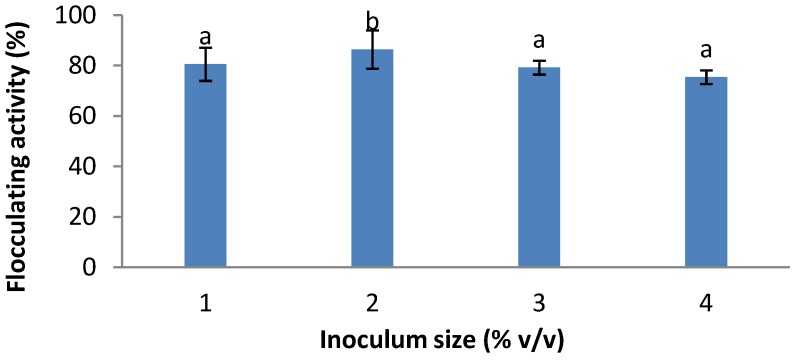
Effects of inoculum size on bioflocculant production by *Cobetia* sp. OAUIFE. Flocculating activities with different letters are significantly different (*p* < 0.05) from each other.

The result of the effect of inoculum size on bioflocculant production is as shown in Figure 1. Inoculum sizes of 0.5 mL, 1.0 mL, 1.5 mL and 2.0 mL in 50 mL production medium were investigated, representing 1%, 2%, 3% and 4% (v/v) respectively. Inoculum size of 2% (v/v) resulted in the highest flocculating activity (86.4%). Further increases in inoculum size resulted in decreases in flocculating activities. As a result, an inoculum size of 2% was used for all subsequent experiments.

**Figure 2:**
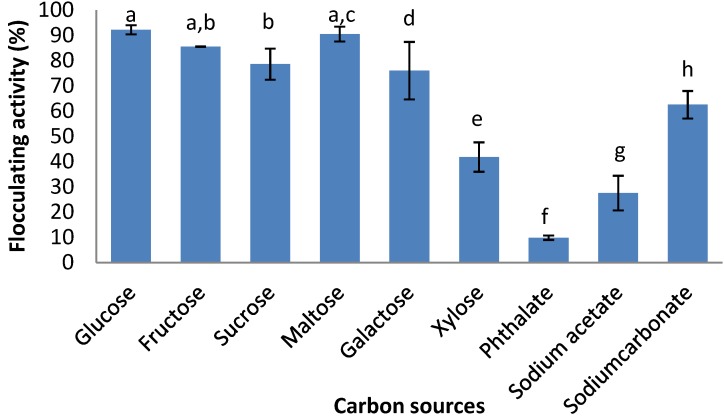
Effect of carbon sources on bioflocculant production of *Cobetia* sp. OAUIFE. Flocculating activities with different letters are significantly different (*p* < 0.05) from each other.

Figure 2 shows the effects of various carbon (20 g/L) sources on bioflocculant production. The bacteria effectively utilized all organic carbon sources for bioflocculant production with flocculating activities of more than 70% except for xylose. The bacteria poorly utilized all the inorganic carbon sources except for sodium carbonate, which resulted in flocculating activity of 62.5%. Glucose and maltose were favourable carbon sources for the test bacteria, with flocculating activities of 92.2% and 90.5% respectively. Glucose was used for all subsequent cultures.

**Figure 3:**
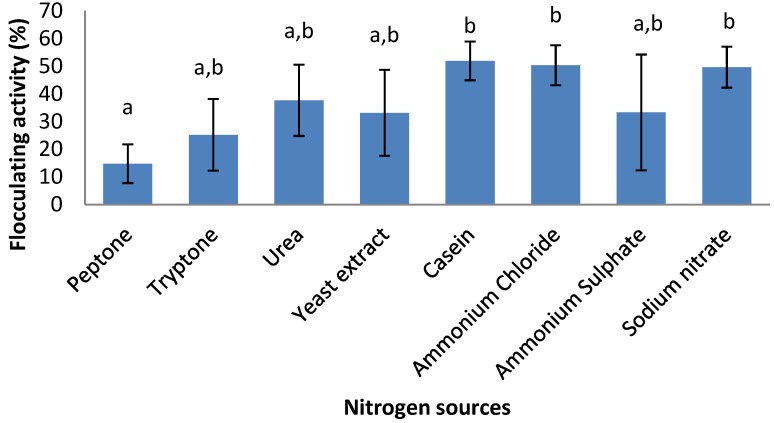
Effect of nitrogen sources on bioflocculant production of *Cobetia* sp. OAUIFE. Flocculating activities with different letters are significantly different (*p* < 0.05) from each other.

The bacteria utilized various nitrogen sources (1.2 g/L) resulting in production of bioflocculant with flocculating activities ranging from 14.8% to 51.9% (Figure 3). Organic nitrogen sources were poorly utilized with virtually all resulting in flocculating activities of less than 50% except for casein which along with the inorganic nitrogen sources ammonium chloride and sodium nitrate resulted in flocculating activity of about 50%.

**Figure 4:**
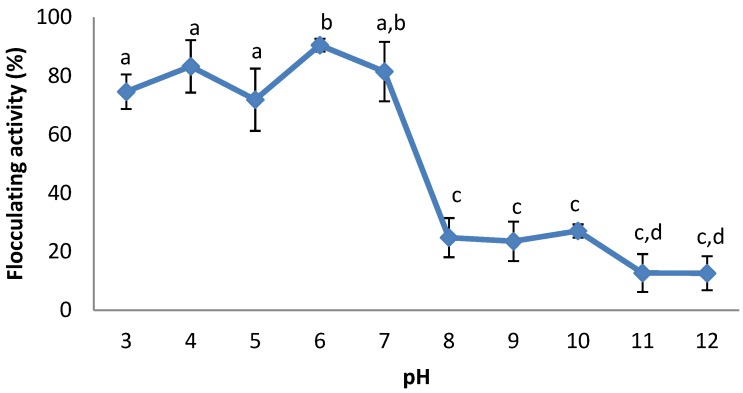
Effect of initial pH on bioflocculant production of *Cobetia* sp. OAUIFE. Flocculating activities with different letters are significantly different (*p* < 0.05) from each other.

The effect of initial medium pH on bioflocculant production was investigated and reported as shown in Figure 4. Bioflocculant was produced at pH ranges of 3–7, with the highest flocculating activity (90.5%) observed at a weakly acidic pH of 6. Alkaline pH was unfavourable for the production of bioflocculant by the bacteria.

All the metal ions significantly stimulated bioflocculant production resulting in very high flocculating activities, except for Fe^3+^ which completely inhibited bioflocculant production as evident from the zero flocculating activity. The extent of preference of the metal ion for bioflocculant production followed the order Mn^2+^>K^+^>Na^+^>Ca^2+^>Li^+^>Mg^2+^>Al^3+^ (Table 1).

**Table 1:** Effect of metal ions on bioflocculant production of *Cobetia* sp. OAUIFE.

METAL IONS	FLOCCULATING ACTIVITY (%)
Na^+^	93.61 ± 1.27 ^a^
K^+^	94.36 ± 0.51 ^a^
Li^+^	91.35 ± 1.69 ^a^
Ca^2+^	92.20 ± 1.83 ^a^
Mn^2+^	95.02 ± 1.17 ^a^
Mg^2+^	90.90 + 0.78 ^a^
Al^3+^	77.41 ± 1.94 ^a^
Fe^3+^	0.00 ^b^

Flocculating activities with different letters are significantly different (*p* < 0.05) from each other.

**Figure 5:**
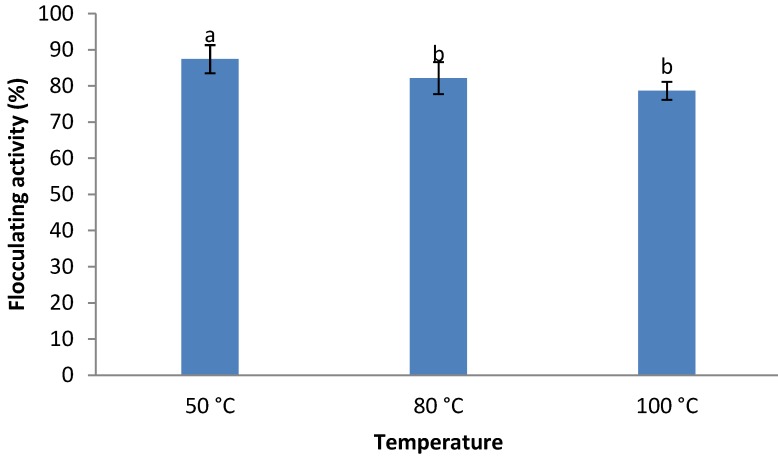
Effect of heat on the flocculating activity of the crude bioflocculant from *Cobetia* sp. OAUIFE. Flocculating activities with different letters are significantly different (*p* < 0.05) from each other.

With regards to the effect of heat of the crude bioflocculant, heating of the bioflocculant culture broth at 100 °C for 25 min revealed a residual flocculating activity of 78%, while heating for the same period at 50 °C and 80 °C resulted in residual flocculating activities of 87% and 82%, respectively (Figure 5).

**Figure 6:**
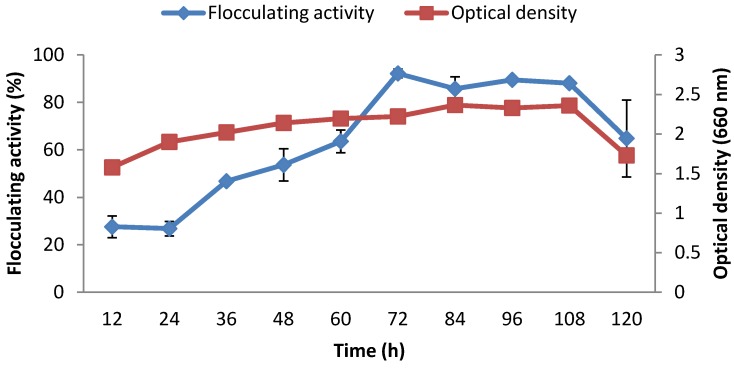
Growth and bioflocculant production curves of *Cobetia* sp. OAUIFE.

Figure 6 reveals the profile of the time course experiment. During the exponential growth phase, flocculating activity increased steadily, until it peaked after 72 hour (with a flocculating activity of 92.2%) beyond which flocculating activity started to decline. It was also observed that the optical density of the culture with time ran almost parallel with the flocculating activity curve.

The biochemical analysis of the purified bioflocculant (Table 2) showed that it was mainly composed of uronic acid (93%), protein (5%) and neutral sugar (1.8%).

This study revealed that an inoculum size of 2% (v/v) of the pre-culture resulted in the highest flocculating activity (86.4%) and further increases in inoculum size resulted in decreases in flocculating activities. A similar observation was made by Salehizadel and Shojaosadati [4]. As a result, an inoculum size of 2% was used for all subsequent experiments. Inoculum size affects not only the cell growth of microorganisms but also their product formation [25,26]. Some studies have reported correlations between inoculum size and bioflocculant production by some organisms. Zhang *et al.* [13] obtained maximum flocculating activity with an inoculum size of 1% (v/v), while Jang *et al*. [27] and Wang *et al.* [17] observed best flocculation with an inoculum size of 5% (v/v). 

**Table 2:** Percentage composition of purified bioflocculant produced by *Cobetia* sp. OAUIFE.

Component	% (w/w)
Protein	5
Neutral sugar	1.8
Uronic acid	93

With respect to the effect of carbon sources on bioflocculant production, the bacteria effectively utilized all organic carbon sources for bioflocculant production with flocculating activities of more than 70%, except for xylose. The bacteria poorly utilized all the inorganic carbon sources except for sodium carbonate, which resulted in flocculating activity of 62.5%. Glucose and maltose were favourable carbon sources for the test bacterium, with flocculating activities of 92.2% and 90.5%, respectively. Glucose was the most preferred and least expensive carbon source, and its preference for bioflocculant production has been reported [9,14,18,28,29,30].

It has been well documented that nitrogen source(s) are important nutrient factor(s) that enhance bioflocculant production [9,14,29]. Most microorganism utilise either organic or inorganic nitrogen sources, or both, to produce bioflocculant. Liu *et al.* [9] reported that *Chryseobacterium daeguense* W6 produces bioflocculant by using organic nitrogen sources for which tryptone was the most preferred, resulting in flocculating activity of more than 90%, while all inorganic nitrogen sources (sodium nitrate, ammonium sulphate and ammonium nitrate) resulted in poor flocculating activity. In another study, Piyo *et al.* [15] observed that *Bacillus* sp. Gilbert utilised an inorganic nitrogen source—ammonium chloride effectively, to produce bioflocculant with a flocculating activity of 91%, while organic nitrogen sources like urea and peptone were poorly utilised. For this current study all the tested organic and inorganic nitrogen sources were poorly utilised individually by the test bacterium as sole nitrogen sources, but the combination of urea, yeast extract and ammonium sulphate, as composed in the production medium resulted in high flocculating activity of 92.2% (Figure 2).

Documented reports revealed that initial pH values required for bioflocculant production differ for different organisms. For example *Bacillus* sp. PY 90 and *Streptomycetes griseus* produced bioflocculant under acidic conditions [31,32], while *Virgibacillus* sp. Rob and *Bacillus licheniformis* X14 optimally produced bioflocculant under alkaline pH [8,14], and *Paecilomyces* sp. [33], and *Halomonas* sp. OKOH [2], produce bioflocculant optimally under neutral pH. In the current study, the bacteria produced bioflocculant well in the range of pH 3–7, with the highest flocculating activity (90.5%) observed at a weak acidic pH of 6. Alkaline pH was unfavourable for the production of bioflocculant by the bacterium. Similar observations were reported for bioflocculant production by *Chryseobacterium daeguense* W6 [9], *Klebsiella mobilis* [17], and *Serratia ficaria* [18]. 

Metal ions either stimulate or inhibit bioflocculant production [8,9,34]. Among the mechanisms proposed for stimulation are: (1) neutralization and stabilization of the residual charge of functional group on the bioflocculant by the metal ions [35], and (2) increase in ionic strength of the suspension solution as a result of addition of metal ion; thereby, decreasing electrostatic forces of the suspended particles [36]. 

Bioflocculant production was stimulated by Na^+^, K^+^, Li^+^, Ca^2+^, Mg^2+^ and Mn^2+^ resulting in flocculating activities of over 90%, while Fe^3+^ inhibited bioflocculant production completely. Optimal flocculating activity (95%) was observed with Mn^2+^. In a similar report, Feng and Xu [34], reported that bioflocculant production by *Bacillus* sp. BF3-3 was stimulated by Al^3+^, Mg^2+^ , Ca^2+^, K^+^ and Na^+^ but inhibited by Fe^3+^. In another report, bioflocculant production was stimulated in the presence of Mg^2+^, Ca^2+^, K^+^ , Mn^2+^, but was inhibited by Ba^2+^, Fe^3+^, Al^3+^ [9].

Heating up to 100 °C for 25 min does not appear to significantly affect the flocculating activity of the bioflocculant, suggesting it to be very thermostable. The bioflocculant appears to be more stable than most similar compounds reported in literature. He *et al.* [37] reported that heating bioflocculant REA-11 (a purified bioflocculant) to 100 °C causes it to completely lose activity. Another bioflocculant (As-101) lost 50% of its flocculating activity when heated at 100 °C for 15 min [38]. Yokoi *et al.* [31] also reported that almost all residual flocculating activity was lost when the bioflocculant of *Bacillus* PY-90 was heated at 100 °C for 40 min. The thermal stability of this bioflocculant might be a result of the polysaccharide backbone in its structure, as is suggested elsewhere [39]. From the analysis of the chemical composition of the purified bioflocculant (2.1 g/L), the high content of uronic acid in the bioflocculant will provide large quantities of carboxyl and hydroxyl functional groups in the polysaccharide backbone, which will interact with each other to create a considerable amount of hydrogen bonds within the molecule, necessary for thermal stability of the bioflocculant.

During the exponential growth phase, flocculating activity increased steadily, until it peaked after 72 h (flocculating activity of 92.2%), beyond which flocculating activity started to decline. This trend suggests that the bioflocculant was produced by biosynthesis, and the decline after 72 h could be due to cell autolysis and/or presence of a bioflocculant-degrading enzyme [19,40]. A similar finding was reported for bioflocculant production by *Serratia ficaria* [18]; however, several studies reported different culture times for maximum bioflocculant production by different bacteria. For example, in *Bacillus firmus*, bioflocculant production peaked after 33 h [41]. In *Agrobacterium* sp. M-503, production peaked after 48 h [42]; while, for *Vagococcus* sp. W31, production peaked after 60 h [19]. Also, in *Phormidium* strain J-1, production peaked after 96 h [43]; while, for *Halomonas* sp. OKOH [2], and *Bacillus* sp. Gilbert [15], maximum flocculating activities were attained in 135 h and 240 h respectively. It was also observed that the bacterial growth profile ran almost parallel with bioflocculant production (Figure 6), suggesting that the bioflocculant production was by biosynthesis during the growth phase of the bacteria. 

## 4. Conclusions

This study has shown *Cobetia* sp. OAUIFE to be a source of new thermostable acidic polysaccharide bioflocculant. The bioflocculant showed excellent flocculating activity of kaolin suspensions, and was optimally produced with glucose as a carbon source and urea, yeast extract and ammonium sulphate combined as nitrogen sources, in the presence of Mn^2+^ with weak acidic pH 6 . This is the first report implicating the *Cobetia* genus in bioflocculant production, the outcomes of which forms the basis of current in-depth ongoing studies on the organism, and we propose that *Cobetia* sp. OAUIFE has immense potential as a producer of bioflocculant that could be of relevance as alternatives to harmful inorganic and synthetic flocculants. 
